# RNase L Suppresses Androgen Receptor Signaling, Cell Migration and Matrix Metalloproteinase Activity in Prostate Cancer Cells

**DOI:** 10.3390/ijms18030529

**Published:** 2017-03-01

**Authors:** Shubham Dayal, Jun Zhou, Praveen Manivannan, Mohammad Adnan Siddiqui, Omaima Farid Ahmad, Matthew Clark, Sahezeel Awadia, Rafael Garcia-Mata, Lirim Shemshedini, Krishnamurthy Malathi

**Affiliations:** Department of Biological Sciences, 2801 W. Bancroft St., University of Toledo, Toledo, OH 43606, USA; Shubham.Dayal@rockets.utoledo.edu (S.D.); Jun.zhou@rockets.utoledo.edu (J.Z.); Praveen.Manivannan@rockets.utoledo.edu (P.M.); Mohammad.Siddiqui@rockets.utoledo.edu (M.A.S.); Omaima.Ahmad@rockets.utoledo.edu (O.F.A.); clark.matt11@gmail.com (M.C.); Sahezeel.Awadia@rockets.utoledo.edu (S.A.); Rafael.GarciaMata@utoledo.edu (R.G.-M.); Lirim.Shemshedini@utoledo.edu (L.S.)

**Keywords:** RNase L, androgen receptor, filamin A, prostate cancer

## Abstract

The interferon antiviral pathways and prostate cancer genetics converge on a regulated endoribonuclease, RNase L. Positional cloning and linkage studies mapped Hereditary Prostate Cancer 1 (*HPC1*) to *RNASEL*. To date, there is no correlation of viral infections with prostate cancer, suggesting that RNase L may play additional roles in tumor suppression. Here, we demonstrate a role of RNase L as a suppressor of androgen receptor (AR) signaling, cell migration and matrix metalloproteinase activity. Using RNase L mutants, we show that its nucleolytic activity is dispensable for both AR signaling and migration. The most prevalent HPC1-associated mutations in RNase L, R462Q and E265X, enhance AR signaling and cell migration. RNase L negatively regulates cell migration and attachment on various extracellular matrices. We demonstrate that RNase L knockdown cells promote increased cell surface expression of integrin β1 which activates Focal Adhesion Kinase-Sarcoma (FAK-Src) pathway and Ras-related C3 botulinum toxin substrate 1-guanosine triphosphatase (Rac1-GTPase) activity to increase cell migration. Activity of matrix metalloproteinase (MMP)-2 and -9 is significantly increased in cells where RNase L levels are ablated. We show that mutations in RNase L found in HPC patients may promote prostate cancer by increasing expression of AR-responsive genes and cell motility and identify novel roles of RNase L as a prostate cancer susceptibility gene.

## 1. Introduction

RNase L is an interferon-regulated endoribonuclease that provides cellular defense against virus infections by targeting diverse RNA substrates [1,2]. Other non-canonical roles of RNase L in regulating barrier function, cellular differentiation, senescence, development of diabetes, lipid storage and demyelination of axons indicate broader roles than the established antiviral functions [3,4,5,6,7]. Genetic association of Hereditary Prostate Cancer 1 (*HPC1*) to *RNASEL* expands the role of RNase L to include tumor suppression [8]. However, beyond the established antiviral effect via nucleolytic function, little is known about the antitumor function of RNase L. Four germline mutations in *HPC1*/*RNASEL* have been identified in hereditary prostate cancer cases: M1I (start codon substitution), E265X (stop codon at 265), 471ΔAAAG (deletion causing a frameshift and stop codon) and R462Q (missense mutation at 462) [8,9,10,11]. The variant RNase L R462Q, which is defective in inducing apoptosis and has a three-fold decrease in enzymatic activity, was reported in 43% of early onset cases of hereditary prostate cancer [12]. However, in some studies, no clear correlation of prostate cancer with RNase L R462Q mutation has been observed indicating heterogeneous disease with more complex etiology involving multiple genes and factors [13,14,15]. Previous studies show that prostate cancer cells depleted of RNase L were resistant to apoptosis by the combined treatment of anti-cancer drugs, TNF-related apoptosis-inducing ligand (TRAIL) and Camptothecin, suggesting that mutations in RNase L may render tumor cells refractory to cell death by conventional therapies [16]. 

RNase L is expressed in all cell types as a latent enzyme. It is activated by a unique and specific oligonucleotide ligand, 2–5A, that is produced from cellular adenosine 5'-triphosphate (ATP) by oligoadenylate synthetase (OAS) and double-strand RNA (dsRNA) during interferon exposure or viral infections [2,17]. In the absence of 2–5A, RNase L exists as an inactive monomer. Binding to the activator, 2–5A, induces conformational change and dimerization to produce an active endoribonuclease which cleaves diverse RNA substrates. The cleaved RNA products amplify interferon production [18], activate inflammasome [19] and promote a switch from autophagy to apoptosis [20]. Recent reports show that RNase L negatively regulates cell migration and downregulates messenger RNAs (mRNAs) for cell adhesion [21,22]. While these established roles of RNase L may contribute to tumor development, they do not provide understanding of how mutations in RNase L predispose to prostate cancer.

RNase L interacts with several cellular proteins like Filamin A, IQ (isoleucineglutamine) motif containing GTPase activating protein 1 (IQGAP1), ligand of numb protein X (LNX), androgen receptor (AR), extracellular matrix (ECM) and cytoskeletal proteins that may provide alternative mechanisms by which it mediates biological functions [3,23,24,25,26]. Recently, we have shown a nuclease-independent role of RNase L in regulating actin dynamics by interacting with an actin-binding protein, Filamin A, to regulate virus entry [3]. RNase L was also reported to interact with AR in breast cancer cells [25]. Filamin A interacts with AR, and a cleaved fragment of Filamin A colocalizes with AR in the nucleus to repress AR-responsive gene expression suggesting important roles for these interactions in regulating androgen signaling [27,28,29]. Several studies demonstrate the importance of microtubules and actin cytoskeleton in shuttling of AR from cytoplasm to the nucleus in cell lines and in clinical samples of prostate cancers [30,31,32]. Considering the requirement of AR to promote prostate cancer and the association of RNase L with genetic predisposition to HPC, we explored the mechanisms that underlie tumor suppression. In this study, we demonstrate the role of RNase L, which did not rely on enzyme activity, as a suppressor of AR signaling, cell migration and matrix metalloproteinase activity. The most prevalent HPC1-associated mutations in RNase L, R462Q and E265X, enhanced AR signaling and cell migration and our studies identify a novel role of RNase L as a prostate cancer susceptibility gene.

## 2. Results

### 2.1. RNase L Negatively Regulates Androgen Signaling

**Figure 1:**
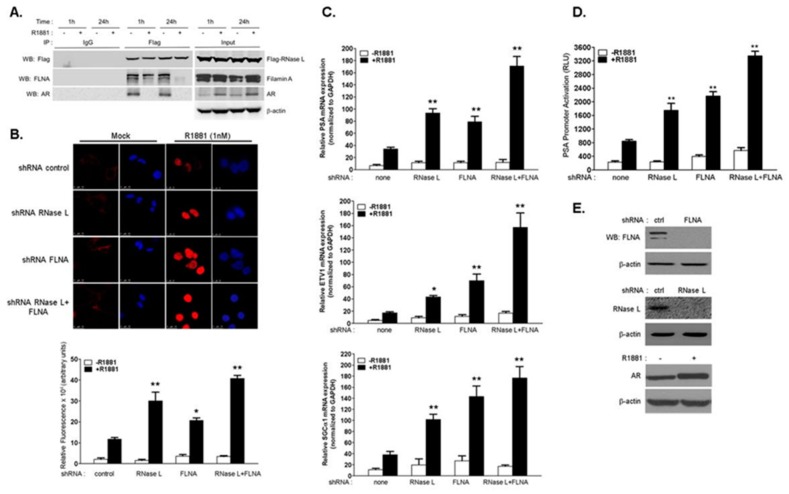
RNase L negatively regulates androgen signaling. (**A**) RNase L interacts with androgen receptor (AR) and Filamin A (FLNA) and dissociates on androgen treatment. LNCaP cells expressing Flag-RNase L were treated with vehicle or R1881 (1 nM) for 1 h or 24 h and immunoprecipitated with Flag-M2 agarose beads or isotype control immunoglobulin G (IgG) beads. The samples were separated on sodium dodecyl sulfate-polyacrylamide gel electrophoresis (SDS-PAGE), and the presence of AR or FLNA was determined using specific antibodies by Western blot analysis (WB). (**B**) Localization of endogenous AR in LNCaP cells expressing short hairpin RNA (shRNA) to knockdown RNase L, Filamin A or both compared to control shRNA. Cells grown in 2% charcoal stripped serum media were treated with vehicle (mock) or R1881 (1 nM) for 24 h, stained with AR antibody followed by Alexa 647-conjugated secondary antibodies and analyzed under confocal microscope. Images are representative of experiments performed in triplicate. AR localization in the nucleus (stained with diamidino-2-phenylindole (DAPI) in blue) was quantitated by measuring fluorescence intensity using Image J software. More than 10 cells (from at least three fields) were analyzed from three independent experiments. RNase L inhibits AR-mediated gene expression. LNCaP cells expressing shRNAs (as in B) were treated or mock-treated and (**C**) quantitative reverse transcriptase-polymerase chain reaction (RT-PCR) of messenger RNA (mRNA) levels of *PSA*, *ETV1* and *sGCα1* was determined and normalized to glyceraldehyde-3-phosphate dehydrogenase (*GAPDH*) mRNA levels. (**D**) Prostate specific antigen (PSA)-luciferase promoter activity was determined 18 h after R1881 treatment and normalized to β-galactosidase levels. Data shown are mean values ± standard deviation (SD) of experiments performed in triplicate from three independent experiments. Student’s *t-*test was used to determine *p*-values. * *p* < 0.01, ** *p* < 0.001 and compared to cells expressing control shRNA and treated with R1881. (**E**) Cell lysates were analyzed on immunoblots for knockdown of RNase L or Filamin A and increase in AR on R1881 treatment normalized to β-actin levels. Scale bar 10 µm; Magnification ×63.

Mutations in RNase L correlate with HPC and RNase L interacts with AR and Filamin A (FLNA) [3,25]. To determine the role of RNase L in HPC, we first examined the effect of androgen, R1881, on the interaction of RNase L with AR and FLNA. Androgen-responsive LNCaP cells were transfected with Flag-RNase L and treated with R1881 (1 nM), and the interaction with AR and FLNA was analyzed by coimmunoprecipitation. In untreated cells, Flag-RNase L interacts with AR and FLNA (Figure 1A). Following treatment with R1881 for 1 h, AR dissociates from Flag-RNase L and there was reduced FLNA associated with Flag-RNase L which decreased further at 24 h. In the absence of ligand, AR remains in the cytoplasm and translocates to the nucleus on binding to androgens to regulate transcription of androgen-responsive genes [33,34]. To determine the impact of RNase L on AR subcellular localization, RNase L was depleted in LNCaP cells using short hairpin RNA (shRNA) and stimulated with R1881 (1 nM) for 24 h and analyzed by confocal microscopy. Increased nuclear AR staining was observed only after R1881 treatment (Figure 1B, top) as quantified by measuring fluorescence intensity from three or more fields from three independent experiments (Figure 1B, bottom). Since RNase L interacts with FLNA in addition to AR, we knocked-down expression of FLNA or both RNase L and FLNA in LNCaP cells (Figure 1E) and stimulated with R1881 for 24 h. Cells lacking FLNA expression showed increased nuclear AR staining which was further increased when both RNase L and FLNA were depleted (Figure 1B). To test if the effect of RNase L on AR nuclear accumulation impacts AR-responsive gene expression, mRNA levels of AR target genes *PSA*, *ETV1* and *sGCα1* were determined in response to R1881 (Figure 1C). The effect of RNase L on AR-transcriptional activity was determined in cells expressing prostate specific antigen (PSA)-luciferase reporter construct, which has copies of AR-response elements fused to luciferase reporter, after stimulating with R1881 (Figure 1D). Increased expression of AR-responsive genes and AR-transcriptional activity correlated with increase in AR nuclear localization in cells lacking RNase L or FLNA and the effect was potentiated in cells lacking both RNase L and FLNA. These results suggest that RNase L negatively regulates androgen signaling and these effects may be mediated, in part, by interaction with FLNA.

**Figure 2:**
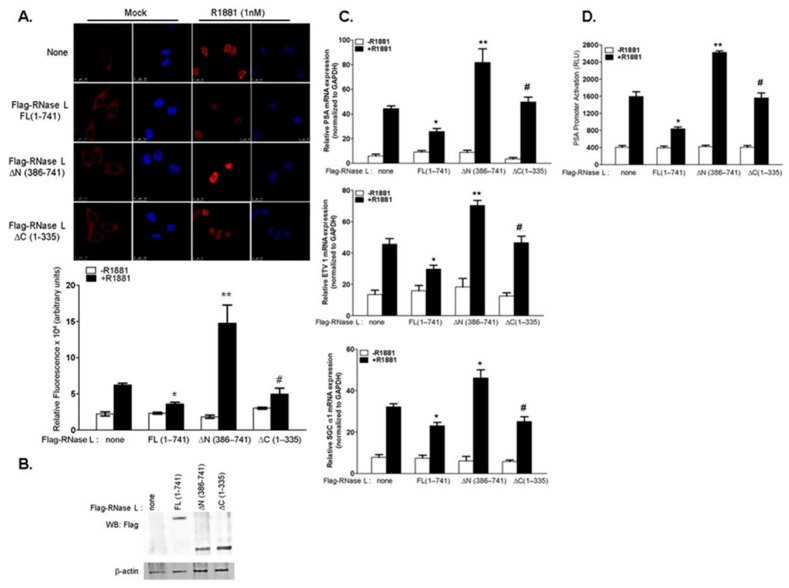
Over-expression of RNase L suppresses AR signaling. (**A**) LNCaP cells expressing full-length Flag-RNase L (FL 1–741), Flag-RNase L lacking N-terminal 385 amino acid residues (ΔN 386–741), Flag-RNase L lacking C-terminal 336–741 amino acid residues (ΔC 1–335) or vector alone (none) were grown in 2% charcoal stripped serum containing media and treated with R1881 or vehicle for 24 h, analyzed under confocal microscope for AR nuclear localization and quantitated as in Figure 1B. (**B**) Cell lysates were analyzed on immunoblots for expression of Flag-RNase L (full-length and truncated proteins) and normalized to β-actin levels. LNCaP cells overexpressing full-length or truncated RNase L mutants as above were treated with R1881 or mock-treated and (**C**) quantitative RT-PCR of mRNA levels of *PSA*, *ETV1* and *sGCα1* was determined and normalized to *GAPDH* mRNA levels. (**D**) PSA-luciferase promoter activity was determined 18 h after R1881 treatment and normalized to β-galactosidase levels. Data shown are mean values ± SD of experiments performed in triplicate from three independent experiments. Student’s *t-*test was used to determine *p*-values. * *p* < 0.01, ** *p* < 0.001, # not significant, and compared to cells expressing vector alone and treated with R1881. Scale bar 10 µm; Magnification ×63.

RNase L has an N-terminal ankyrin repeat domain, a pseudokinase domain in the middle and a C-terminal ribonuclease domain. RNase L binds to its activator, 2–5A, via the ankyrin repeat and pseudokinase domains allowing dimerization which is required for nuclease activity. To identify the region of RNase L that is required for suppression of androgen signaling, we over-expressed full-length Flag-RNase L (FL 1–741), N-terminal 1–335 amino acid residues (lacking nuclease domain (ΔC (1–335)) or C-terminal 386–741 amino acid residues (lacking ankyrin repeats (ΔN (386–741)) or vector alone in LNCaP cells (Figure 2B) and stimulated with R1881 for 24 h. Compared to cells expressing endogenous levels of RNase L (Figure 2, labeled as none), over-expression of RNase L suppressed AR nuclear localization two-fold (Figure 2A) which correlated with decrease in expression of AR-responsive genes (Figure 2C) and AR-transcriptional activity (Figure 2D). Interestingly, the N-terminal domain of RNase L which lacks nuclease activity suppressed androgen signaling, whereas expression of C-terminal domain alone increased AR nuclear localization, mRNA levels of AR target genes as well as AR-transcriptional activity (Figure 2A–D). Taken together, our results show that RNase L suppresses androgen signaling in LNCaP cells and the N-terminal ankyrin repeat domain is required for this effect.

### 2.2. The Effect of RNase L on AR Signaling Is Not Due to Altered AR Stability

**Figure 3:**
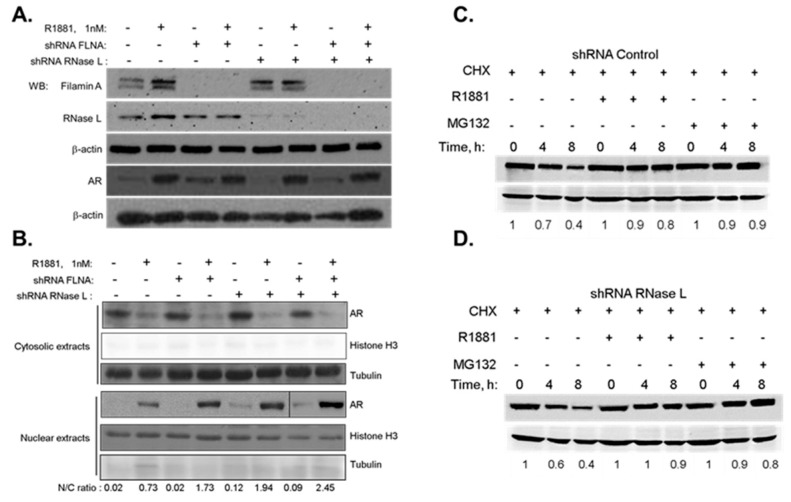
Increased AR localization in the nucleus in RNase L-depleted cells is not due to altered AR stability. LNCaP cells expressing shRNA to knockdown RNase L, FLNA or both and control shRNA were grown in 2% charcoal-stripped serum containing media and treated with vehicle or R1881 and (**A**) Cell lysates were analyzed on immunoblots for knockdown and AR expression levels. (**B**) Cells were fractionated and AR from nuclear (N) and cytosolic (C) extracts were analyzed by immunoblotting with anti-AR, histone H3 (marker for nuclear extract), α-tubulin (marker for cytosolic extract) antibodies. Nuclear-to-cytoplasmic (N/C) ratio of AR protein with or without R1881 was determined by densitometric analysis of band intensities using Image J software. LNCaP cells with control shRNA (**C**) or RNase L shRNA (**D**) in growth medium were treated with cycloheximide (CHX, 50 μg/mL) alone or combined with MG132 (20 μM) or R1881 (1 nM) for 4 h or 8 h. Cell lysates were prepared in 2% SDS and levels of AR were normalized on immunoblots with β-actin and relative changes in AR levels compared to CHX treatment for 0 h (band intensity set as 1) was determined. Similar results were observed in three independent experiments.

Our results show that knockdown of endogenous RNase L increased AR signaling and over-expression of RNase L in LNCaP cells resulted in marked reduction of nuclear AR levels. We analyzed the subcellular distribution of AR in cells with endogenous levels of RNase L and FLNA, knockdown of either RNase L or FLNA and knockdown of both RNase L and FLNA by cellular fractionation following R1881 treatment. We validated the knockdown of RNase L and or FLNA and induction of AR expression in response to R1881 in immunoblots (Figure 3A). Cytosolic and nuclear extracts of cells with and without R1881 treatment were analyzed for AR protein levels. Consistent with our imaging experiments (Figure 1B), treatment of cells with R1881 resulted in translocation of AR from the cytosolic to the nuclear fraction. Quantitation of immunoblots for AR protein shows that depleting RNase L or FLNA increased AR nuclear-to-cytoplasmic ratio (N/C ratio), and depleting both resulted in further increase in nuclear AR levels (Figure 3B).

Due to the inhibitory effect of RNase L on AR nuclear accumulation, we tested the possibility that RNase L may affect AR protein stability. Control and RNase L shRNA expressing LNCaP cells were treated with cycloheximide (CHX) for the indicated times to block *de novo* protein translation and combined with either proteasome inhibitor, MG132, to block AR degradation or R1881 to induce AR protein levels. As demonstrated by other studies, AR protein levels declined in cells treated with CHX alone [35] and the reduced levels were comparable in cells depleted or expressing RNase L (Figure 3C,D). No differences in MG132 stabilized AR was observed in both cells suggesting that RNase L does not alter AR stability.

### 2.3. Hereditary Prostate Cancer 1 (HPC1)-Associated Mutants of RNase L Enhance AR Transcriptional Activity

**Figure 4:**
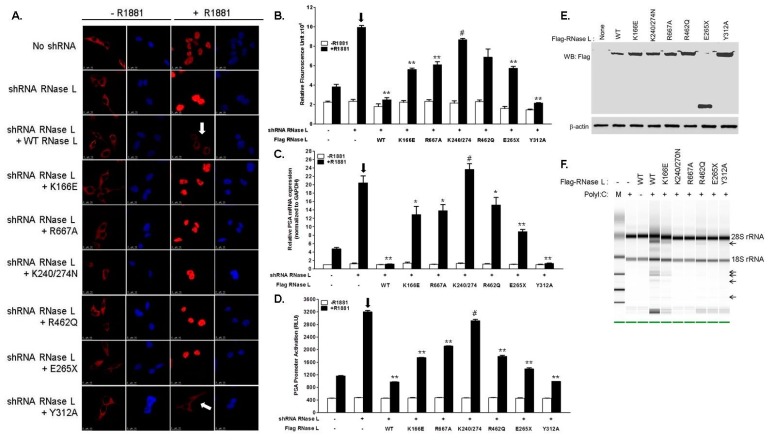
Hereditary Prostate Cancer 1-associated mutants of RNase L enhance AR transcriptional activity. (**A**) Endogenous RNase L-knockdown LNCaP cells were reconstituted with Flag-Wild-type (WT) or mutated RNase L as indicated and treated with vehicle (−R1881) or R1881 (1 nM) for 24 h. AR nuclear localization was analyzed under confocal microscope; and (**B**) quantitated by measuring fluorescence intensity using Image J software as described in Figure 1B. White arrows point to lack of nuclear localization in WT and RNase L Y312A mutant expressing cells. AR transcriptional activity was monitored in LNCaP cells reconstituted with WT and RNase L mutants treated with vehicle or R1881 by (**C**) quantitative RT-PCR of mRNA levels of *PSA*, *ETV1* and *sGCα1* normalized to *GAPDH* mRNA levels. (**D**) PSA-luciferase promoter activity 18 h after R1881 treatment and normalized to β-galactosidase levels. Data shown are mean values ± SD of experiments performed in triplicate from three independent experiments. Student’s *t-*test was used to determine *p*-values. * *p* < 0.01, ** *p* < 0.001, # not significant, and compared to RNase L knockdown cells (shown with arrow on graphs) treated with R1881. (**E**) Cell lysates were analyzed for expression of Flag-RNase L (WT) and mutants as indicated using anti-Flag antibodies and normalized to β-actin levels. (**F**) HeLa cells were transfected with Flag-RNase L (WT) and RNase L mutants and enzyme activity was determined by monitoring rRNA cleavage (shown by arrows) as determined in RNA chips by transfecting cells with PolyI:C (2 μg/mL) and isolation of total RNA. Scale bar 10 µm, Magnification ×63.

To understand how mutations in RNase L contribute to HPC, we examined the effect of HPC1-associated RNase L mutants on androgen signaling. To further address if RNase L enzyme activity is required for androgen signaling, other mutations in RNase L were generated by site-directed mutagenesis of Flag-RNase L construct including R667A (nuclease-dead), K166E (reduced enzyme activity), K240/274N (defective 2–5A binding and lacks enzyme activity), Y312A (defective 2–5A binding and lacks enzyme activity). Expression of Flag-RNase L Wild type (WT) and mutant proteins is shown in immunoblots (Figure 4E). RNase L activity of the WT and mutant constructs was determined by rRNA cleavage in response to PolyI:C transfection in HeLa cells which lack detectable RNase L activity (Figure 4F) [36,37]. As endogenous RNase L may mask some of the effects of mutants we express, we used a knockdown/rescue approach in which endogenous RNase L levels were depleted in LNCaP cells with shRNA targeting the 3′-UTR (Figure 1E) and reconstituted with Flag-RNase L (WT) or RNase L mutants and stimulated with R1881 for 24 h. We then monitored the subcellular localization of AR by confocal microscopy (Figure 4A) and quantitated the amount of nuclear AR (Figure 4B). Expression of WT RNase L suppressed nuclear translocation of AR as we observed in Figure 2. However, cells expressing HPC1-associated mutants, R462Q and E265X showed increased AR in the nucleus. Since both mutants compromised RNase L enzyme activity, we tested other RNase L mutants which had reduced activity (K166E), lacked enzyme activity due to defect in binding the activator 2–5A (K240/274N and Y312A) or in the nuclease domain (R667A) to determine if enzyme activity of RNase L was required for androgen signaling. In contrast with other mutants, RNase L Y312A, which is defective in 2–5A binding and lacks enzyme activity, was able to suppress nuclear AR localization like WT RNase L (Figure 4A, arrows). We did not observe any change in AR subcellular localization in the absence of R1881. The increase in nuclear AR correlated with mRNA levels of AR target genes and AR-transcriptional activity (Figure 4C,D). Together, these results suggest that HPC1-associated RNase L mutants may contribute to HPC by regulating androgen signaling. Further, the effect of RNase L on androgen signaling appears to be independent of nucleolytic functions (Figure 4F).

### 2.4. Cell Migration Is Increased in Cells with Reduced Levels of RNase L

**Figure 5:**
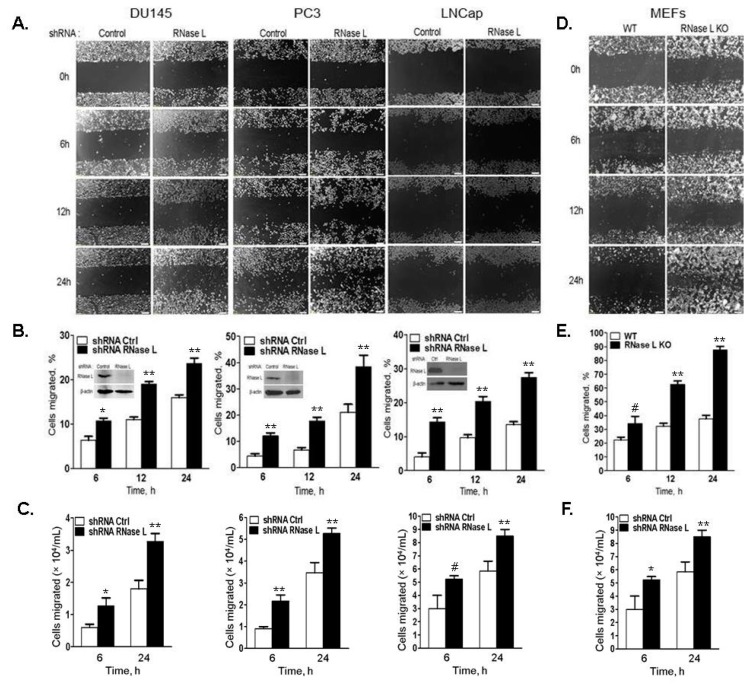
Cell migration is increased in cells with reduced RNase L levels. DU145, PC3 or LNCaP cells expressing control or RNase L shRNA (**A**–**C**) and WT or RNase L KO mouse embryonic fibroblasts (MEFs) (**D**–**F**) were grown to confluence. (**A**,**D**) The cell monolayer was scratched and wound closure was imaged under phase-contrast microscope at indicated times. (**B**,**E**) Cells migrated (%) to close the wound was quantitated by Image J software. Knock-down of RNase L on immunoblots is shown as inset in (**B**). (**C**,**F**) Transwell chambers coated with fibronectin were used to measure cell migration at 6 h and 24 h in response to growth medium with 10% serum by counting cells that migrated to the lower surface of the filters from experiments performed in triplicate. Data shown are mean values ± SD of experiments performed in triplicate from three independent experiments. Student’s *t-*test was used to determine *p*-values. * *p* < 0.01, ** *p* < 0.001, # not significant, and compared to cells expressing control shRNA (**A**–**C**) and compared to WT MEFs (**D**–**F**). Scale bar 100 µm, Magnification ×10.

Our published observation that RNase L interacts with the actin-binding protein, FLNA, to regulate actin dynamics [3] suggests that RNase L may have additional roles in HPC besides regulating androgen signaling. The ability of RNase L to modulate actin cytoskeleton prompted us to explore whether RNase L affected cell migration in prostate cancer cells. To investigate the role of RNase L in prostate cancer cell motility, RNase L levels were knocked-down using shRNA in DU145, PC3 and LNCaP cells and compared to cells expressing endogenous RNase L levels (expressing control non-targeting shRNA) (Figure 5B, inset). Confluent monolayer of cells in serum-free media were scratched and replaced with growth media. Cell migration to close the wound was imaged (Figure 5A) and quantitated (Figure 5B) over time as indicated. In all three prostate cancer cells, depletion of RNase L had marked effect (1.5–2-fold) in enhancing cell migration and the effect was most significant at 24 h experimental endpoint. No significant difference in cell proliferation was observed between control and knockdown cells over the 24 h time course of the experiment (data not shown). The difference in cell migration was quantitated by transwell cell migration assays in response to serum (Figure 5C). Consistent with scratch wound healing assays, RNase L knockdown cells migrated significantly more (1.5–2-fold) through fibronectin-coated filters in response to serum.

**Figure 6:**
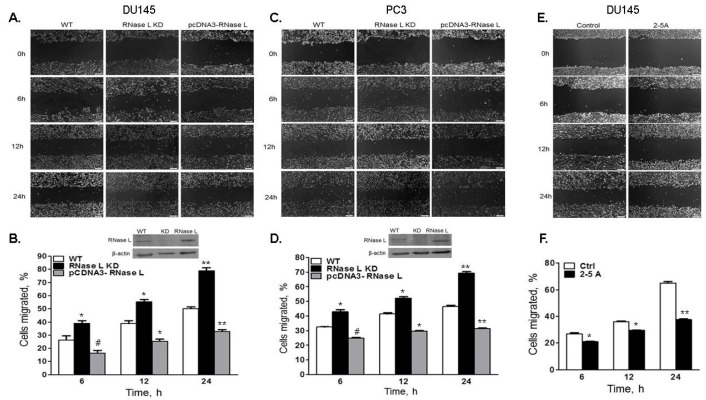
Over-expression or activation of RNase L inhibits cell migration. DU145 (**A**,**B**) or PC3 (**C**,**D**) cells expressing control shRNA and expressing endogenous levels of RNase L (WT), shRNA to knockdown RNase L (RNase L KD) or overexpressing RNase L after knockdown of endogenous RNase L (pcDNA3-RNase L) were grown to confluence. (**A**,**C**) Cell monolayers were scratched and wound closure was imaged under phase-contrast microscope at indicated times. (**B**,**D**) Cells migrated (%) to close the wound was quantitated by Image J software. RNase L protein levels were analyzed on immunoblots using anti-RNase L antibody and shown as inset in B and D. Data shown are mean values ± SD of experiments performed in triplicate from three independent experiments. Student’s *t-*test was used to determine *p*-values. * *p* < 0.01, ** *p* < 0.001, # not significant, and compared to control cells expressing endogenous levels (WT) of RNase L. (**E**) DU145 cells were mock transfected (control) or transfected with 2–5A (10 μM) to activate RNase L. The monolayer was scratched and wound closure was imaged under phase-contrast microscope at indicated times. (**F**) Cells migrated (%) to close the wound was quantitated by Image J software. Data shown are mean values ± SD of experiments performed in triplicate from three independent experiments. Student’s *t-*test was used to determine *p*-values. * *p* < 0.01, ** *p* < 0.001, and compared to mock-treated (control) cells (**E**,**F**). Scale bar 100 µm, Magnification ×10.

To further demonstrate that the increased cell migration was due to lack of RNase L, we used WT and *Rnase l^−/−^* (RNase L KO) primary mouse embryonic fibroblasts (MEFs) and monitored wound closure by wound healing assays and cell migration in transwell assays. Migration of RNase L KO MEFs was enhanced 2–3-fold in response to serum or fibronectin compared to WT MEFs (Figure 5D–F). In addition, we reconstituted RNase L expression in DU145 or PC3 RNase L knockdown cells and compared wound closure in cells with endogenous levels of RNase L, depleted of RNase L or over-expressing RNase L (Figure 6A,C). Expression of RNase L is shown in immunoblots as inset in Figure 6B,D. At 24 h experimental endpoint wound closure was 47% in DU145 cells with endogenous RNase L (WT), 76% in RNase L-depleted cells and 29% in over-expressing cells. In PC3 cells, endogenous RNase L expressing cells (WT) showed 45%, RNase L-depleted cells showed 68% and over-expressing cells showed 27% wound closure. To determine the consequence of RNase L activation on cell migration, DU145 cells were transfected with activator, 2–5A which causes RNase L dimerization, or mock transfected and wound closure was imaged and quantitated (Figure 6E,F). 2–5A treatment of cells decreased migration of DU145 cells by 48% compared to mock transfected cells. Taken together, these results demonstrate that RNase L inhibits cell migration in prostate cancer and primary cells. Furthermore, activation of RNase L by 2–5A, which results in conformational change and dimerization of RNase L, also suppresses cell migration.

### 2.5. Hereditary Prostate Cancer-Associated Mutants of RNase L Promote Cell Migration

**Figure 7:**
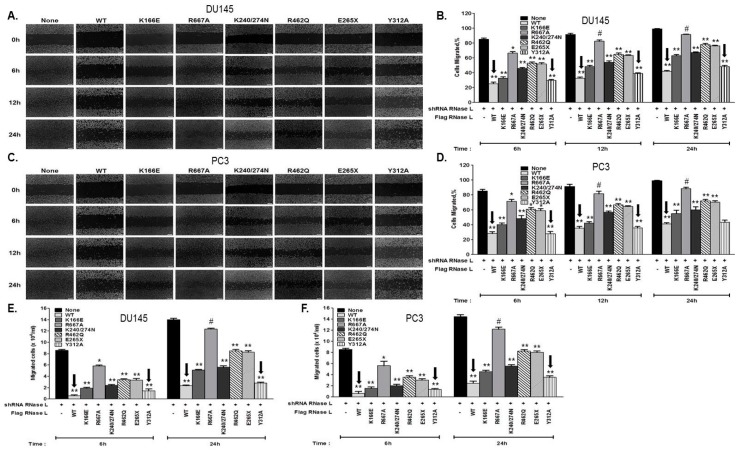
Hereditary prostate cancer-associated mutants of RNase L promote cell migration. Endogenous RNase L was knocked down in DU145 or PC3 cells and reconstituted with Flag vector (none), Flag-RNase L (WT) or Flag-RNase L mutants as indicated. (**A**,**C**) Cell monolayers were scratched and wound closure was imaged under phase-contrast microscope at indicated times. (**B**,**D**) Cells migrated (%) to close the wound was quantitated by Image J software. (**E**,**F**) Transwell chambers coated with fibronectin were used to measure cell migration at 6 h and 24 h in response to growth medium with 10% serum by counting cells that migrated to the lower surface of the filters from experiments performed in triplicate. Arrows are used to highlight RNase L mutants that suppress migration as WT RNase L. Data shown are mean values ± SD of experiments performed in triplicate from three independent experiments. Student’s *t-*test was used to determine *p*-values. * *p* < 0.01, ** *p* < 0.001, # not significant, and compared to cells expressing shRNA to knockdown RNase L. Scale bar 100 µm, Magnification ×10.

We have shown that RNase L suppresses AR signaling and cell migration. Because the most common RNase L mutations associated with HPC enhance AR signaling, we investigated whether these RNase L mutants also affect cell migration. To further explore if RNase L enzyme activity is required for cell migration, we also tested the RNase L mutants we generated that have reduced enzyme activity (K166E) or lack enzyme activity due to mutation in the nuclease domain (R667A) or defect in binding the activator, 2–5A (K240/274N, Y312A). Loss of heterozygosity of RNase L has been observed in HPC1 prostate tumors [8]. Therefore, we depleted RNase L in DU145 and PC3 cells and reconstituted with vector alone (none), Flag-RNase L (WT) or Flag-RNase L mutants as indicated (Figure 7A–F). Confluent monolayers of cells were scratched and wound closure was imaged (Figure 7A,C) and quantitated (Figure 7B,D). Differences in cell migration between cells expressing various RNase L mutants were also quantitated by transwell assays through fibronectin coated filters in response to serum (Figure 7E,F). Consistent with our observations, RNase L knockdown cells showed increased migration and expression of WT RNase L suppressed cell migration. HPC1-associated RNase L mutants, R462Q and E265X, showed increase in cell migration compared to cells expressing WT RNase L (shown by arrows in Figure 7B,D–F). RNase L K166E, which has reduced enzyme activity, R667A, which is nuclease-dead, and K240/274N which lacks enzyme activity showed 1.5–2-fold increase in migration compared to WT expressing cells. In contrast with other RNase L mutants which lack enzyme function, the Y312A mutant which also lacks enzyme activity due to its inability to bind 2–5A, suppressed cell migration (shown by arrows in Figure 7B,D–F). This effect was comparable to WT RNase L indicating that different regions of RNase L, presumably through interacting proteins, may contribute to cell migration rather than enzyme activity.

### 2.6. RNase L-Depletion Increases Cell Attachment and Cell Spreading

**Figure 8:**
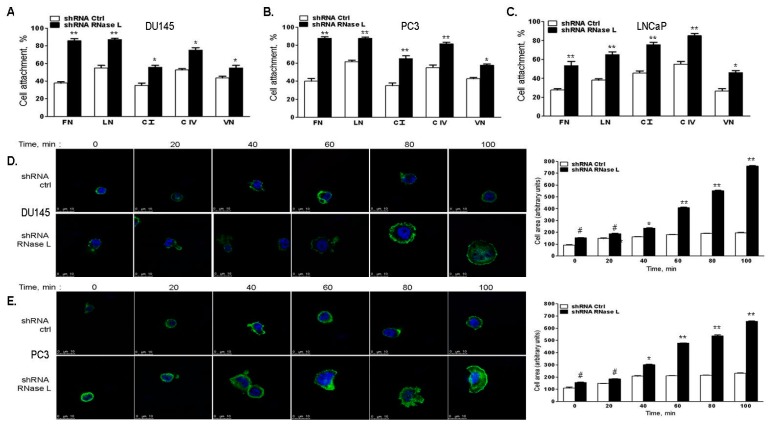
RNase L depletion increases cell attachment to extracellular matrix substrates and cell spreading. (**A**) DU145; (**B**) PC3; or (**C**) LNCaP cells expressing control shRNA or RNase L shRNA were allowed to attach to wells coated with 10 μg/mL each of fibronectin (FN), laminin (LN), collagen I (C I), collagen IV (C IV) or vitronectin (VN) for 1 h. Plates were washed and attached cells were stained. Cell attachment (%) was determined from means ± SD of experiments performed in triplicate from three separate experiments. (**D**) DU145; or (**E**) PC3 expressing control shRNA or RNase L shRNA were allowed to attach and spread on surfaces coated with fibronectin (10 μg/mL) at various time points, fixed and F-actin was labeled with Alexa 488-labeled phalloidin and imaged by confocal microscope. Areas of individual cells from at least 30 measurements were determined by Image J software. Data shown represent the mean of cell area ± standard error of mean (SEM) of three experiments performed in triplicate. Student’s *t*-test was used to determine *p*-values. * *p* < 0.01, ** *p* < 0.001, # not significant, and compared to cells expressing control shRNA. Scale bar 10 µm, Magnification ×63.

Interaction of the cell with extracellular matrix (ECM) causes engagement of specific transmembrane receptors and signaling molecules which modulate cytoskeletal organization resulting in distinct cell morphology, attachment and migration [38]. To characterize the consequence of RNase L on cell attachment, RNase L was knocked-down using shRNA in DU145, PC3 or LNCaP cells and compared to control shRNA expressing cells. Cells were allowed to attach to dishes coated with fibronectin (FN), laminin (LN), collagen I (C I), collagen IV (C IV) or vitronectin (VN) for 1 h. Wells were then washed and attached cells were quantitated by staining. Knockdown of RNase L in all three prostate cancer cell lines tested showed increase in cell attachment to all the ECM substrates, and the difference was 2–2.5-fold greater in response to fibronectin (Figure 8A–C). We monitored DU145 and PC3 cells for both shape and cell spreading after incubation on FN substrates. Expression of RNase L appeared to result in the presence of more rounded cells which spread slowly. In contrast RNase L-depleted cells tend to spread rapidly and the cell area increased in a time-dependent manner (Figure 8D,E). Both the rate and extent of cell spreading were significantly lower at all the time points in RNase L expressing cells. These results suggest that RNase L inhibits cell attachment and cell spreading.

### 2.7. Depletion of RNase L Promotes Integrin Activation and FAK-Src Signaling in Response to Fibronection

**Figure 9:**
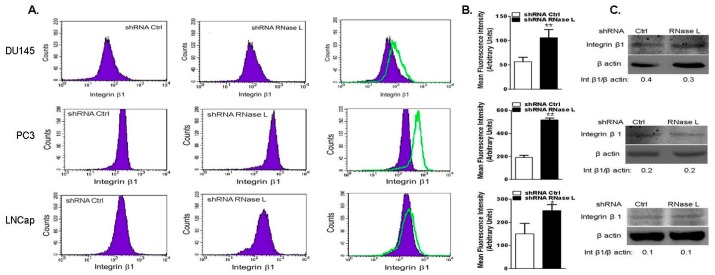
Cells with reduced levels of RNase L exhibit increased surface expression of integrin β1. DU145, PC3 or LNCaP cells expressing control shRNA or RNase L shRNA were plated on fibronectin-coated dishes and analyzed by flow cytometry for surface staining with antibodies against integrin β1 and Alexa-488 conjugated secondary antibodies. (**A**) Representative histograms; and (**B**) bar graphs for the mean fluorescence intensity of at least three independent experiments for integrin β1 are shown. Student’s *t-*test was used to determine *p*-values. * *p* < 0.01, ** *p* < 0.001 and compared to cells expressing control shRNA. (**C**) Expression of integrin β1 in cell lysates was determined on immunoblots and normalized to levels of β-actin.

**Figure 10:**
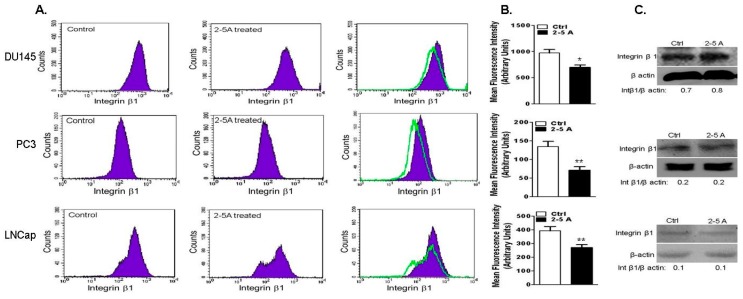
Activation of RNase L reduces surface expression of integrin β1. DU145, PC3 or LNCaP cells were mock-treated (control) or transfected with 2–5A (10 μM) to activate RNase L and plated on fibronectin-coated dishes and analyzed by flow cytometry for surface staining with antibodies against integrin β1 and Alexa-488 conjugated secondary antibodies. (**A**) Representative histograms; and (**B**) bar graphs for the mean fluorescence intensity of at least three independent experiments for integrin β1 are shown. Student’s *t*-test was used to determine *p*-values. * *p* < 0.01, ** *p* < 0.001 and compared to mock-treated cells. (**C**) Expression of integrin β1 in cell lysates was determined on immunoblots and normalized to levels of β-actin.

Integrins are transmembrane receptors that physically link ECM to intracellular actin cytoskeleton [39]. Trafficking and recycling of integrin β1 to the cell membrane and clustering is required for cell attachment, spreading and migration [40]. Clustering of integrins activates signaling events involving focal adhesion kinase (FAK) and Src [41,42]. Since RNase L regulates cell attachment and migration, we investigated if the effects were mediated by integrin-regulated Focal Adhesion Kinase-Sarcoma (FAK-Src) signaling pathways. To assess the role of RNase L in regulating integrin β1 expression and activation, prostate cancer cells depleted of RNase L or WT were allowed to spread on FN and cell suspensions were fixed and incubated with integrin β1 antibodies to label cell surface integrin β1. Flow cytometric analysis showed that cell surface integrin β1 was increased 1.5–3-fold following knockdown of RNase L (Figure 9A–C). No changes in protein levels of integrin β1 were observed between WT and RNase L knockdown cells. Consistent with roles in cell migration, attachment and spreading, RNase L is important for activation of integrin β1. Activation of RNase L with 2–5A which promotes dimerization, inhibits cell migration (Figure 6E,F) and we observed decrease in cell surface integrin β1 expression in DU145, PC3 and LNCaP cells transfected with 2–5A and this effect was not due to decrease in integrin β1 protein levels (Figure 10A–C).

**Figure 11:**
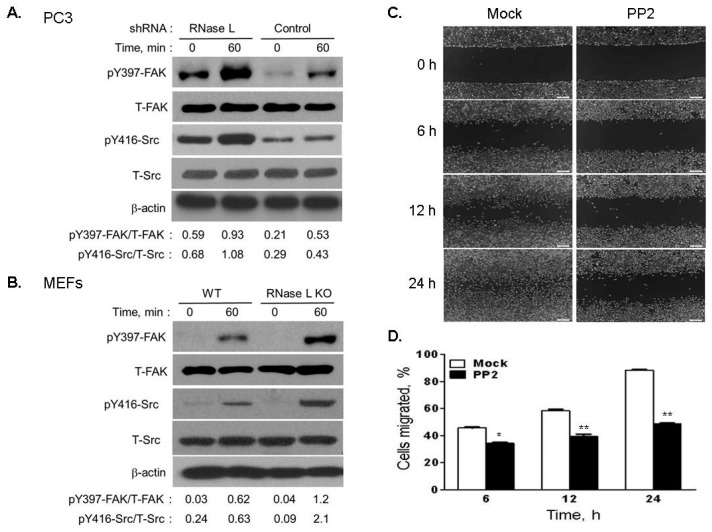
Increased FAK and Src phosphorylation in response to fibronectin in RNase L-depleted cells effects cell migration. (**A**) PC3 cells expressing control or RNase L shRNA; and (**B**) WT and *Rnase l^−/−^* (RNase L KO) MEFs were serum starved and plated on fibronectin-coated plates for indicated times. Phospho-FAK (Y397) and phospho-Src (Y416) was detected in cell lysates using phospho-specific antibodies on immunoblots followed by Western blotting with anti-FAK, anti-Src antibodies and β-actin for loading control. (pY397)-FAK and (pY416)-Src intensity values were normalized to total-FAK and total-Src intensities respectively using Image J software. Representative immunoblots from experiments performed in triplicate are shown. (**C**) PC3 RNase L-depleted cells growing in confluent monolayers were pretreated with vehicle (mock) or Src inhibitor (PP2, 10 μM) for 1 h. Cell monolayers were scratched and wound closure was imaged under phase-contrast microscope at indicated times in growth medium containing PP2 inhibitor. (**D**) Cells migrated (%) to close the wound was quantitated by Image J software. Data shown are mean values ± SD of experiments performed in triplicate from three independent experiments. Student’s *t*-test was used to determine *p*-values. * *p* < 0.01, ** *p* < 0.001, and compared to vehicle-treated (mock) cells. Scale bar 100 µm, Magnification ×10.

FAK is recruited to sites of integrin clustering and activated by autophosphorylation at Y397 which in turn facilitates Src binding [43,44,45]. Phosphorylation of Src at Y416 can lead to formation of FAK-Src signaling complex which acts with downstream regulators including RhoGTPases to control cell shape and turnover of focal adhesion during cell migration [46,47]. We tested FAK activation after integrin β1 clustering in response to FN in PC3 cells with endogenous or knockdown of RNase L and in WT and RNase L KO MEFs. FAK Y397 phosphorylation was increased 43% in PC3 RNase L knockdown cells and 48% in RNase L KO MEFs compared to PC3 control or WT MEFs, respectively (Figure 11A,B). Src is activated by binding to pY397-FAK and we observed a corresponding 60% increase in pY416 Src in PC3 RNase L knockdown cells and 70% in RNase L KO MEFs compared to PC3 control or WT MEFs respectively (Figure 11A,B). In PC3 RNase L knockdown cells, we observed increase in basal levels of phospho-FAK and phospho-Src which increased further on FN stimulation. 

To further demonstrate that Src activity is involved in cell migration, RNase L-depleted PC3 cells were treated with Src inhibitor, PP2, and monolayers were scratched and cell migration to close the wound was imaged and quantitated. In mock-treated cells 85% of wound closure was observed at 24 h compared to 43% in PP2-treated cells (Figure 11C,D). These results suggest that integrin clustering in response to FN in RNase L-depleted cells was significantly enhanced over control cells which in turn reflected in increased phosphorylation of FAK and Src which effects cell migration.

### 2.8. Increased Rac1 Activity Mediates Enhanced Cell Migration in RNase L-Depleted Cells

**Figure 12:**
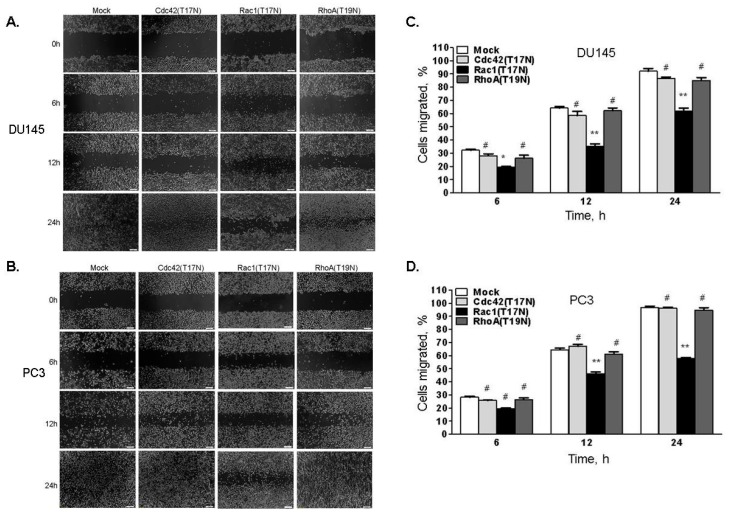
Ras-related C3 botulinum toxin substrate 1 (Rac1) mediates enhanced cell migration in RNase L-depleted cells. RNase L shRNA expressing (**A**) DU145 cells; or (**B**) PC3 cells were transfected with empty vector (mock), dominant negative Cdc42 (T17N), dominant negative Rac1 (T17N) or dominant negative RhoA (T19N) expressing plasmids. After 24 h, cell monolayers were scratched and wound closure was imaged under phase-contrast microscope at indicated times. (**C**,**D**) Cells migrated (%) to close the wound was quantitated by Image J software. Data shown are mean values ± SD of experiments performed in triplicate from three independent experiments. Student’s *t-*test was used to determine *p*-values. * *p* < 0.01, ** *p* < 0.001, # not significant, and compared to RNase L-depleted cells mock transfected with empty vector. Scale bar 100 µm, Magnification ×10.

**Figure 13:**
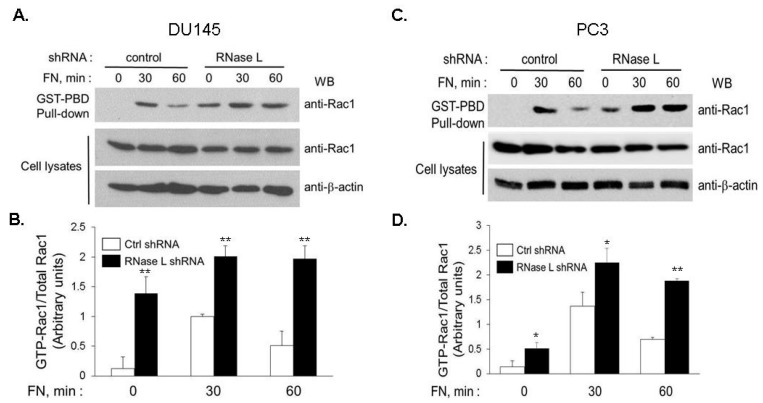
Rac1 activity in response to fibronectin is increased in RNase L-depleted cells. (**A**) DU145; or (**C**) PC3 cells expressing control or RNase L shRNA were plated on fibronectin-coated dishes for indicated times. Cell lysates were incubated with agarose-immobilized GST-PAK1 binding domain (GST-PBD) and co-precipitated proteins were subject to immunoblotting with anti-Rac1 antibodies to detect the amount of GTP-bound Rac1 (active Rac1) and compared to expression of Rac1 in cell lysates. Representative immunoblots from experiments performed in triplicate are shown. (**B**,**D**) Activity of Rac1 was quantitated by comparing the intensities of active Rac1 with those of total Rac1 in each lane using Image J software. Data are mean values ± SEM expressed as increase in Rac1 activity from three independent experiments. Student’s *t-*test was used to determine *p*-values. * *p* < 0.01, ** *p* < 0.001, and compared to control shRNA expressing cells.

Signaling pathways triggered by integrins regulate FAK-Src activity and involve Rho GTPases which are crucial for remodeling cytoskeleton and cell mobility [48,49]. To investigate which of the Rho family, GTPases-RhoA, Cdc42 or Rac1, mediate RNase L-dependent migration, RNase L-depleted DU145 or PC3 cells were transfected with empty vector (mock), or dominant-negative forms of RhoA (T19N), Cdc42 (T17N) or Rac1 (T17N) [50]. Expression of the proteins on immunoblots is shown in Figure S1. Monolayers of cells were scratched and wound closure was imaged and quantitated at indicated times (Figure 12A–D). Expression of the dominant-negative Rac1 significantly inhibited migration of RNase L-depleted cells whereas RhoA (T19N) and Cdc42 (T17N) had marginal effects. To examine the effect of reduced RNase L levels on Rac1 activity in response to FN, we measured Ras-related C3 botulinum toxin substrate 1 (Rac1) activity by precipitating active GTP-bound Rac1 with GST-PAK-binding domain (GST-PBD) and estimating the abundance relative to total levels of Rac1 protein. In both DU145 (Figure 13A,B) and PC3 (Figure 13C,D), adhesion to FN stimulated a rapid and transient increase in Rac1 activity which peaked at 30 min and declined by 1 h. In contrast, RNase L knockdown cells had higher basal level of Rac1 which increased further on FN stimulation and was sustained at 1 h (Figure 13A–D). Our data support the observation that increased integrin and FAK-Src signaling in response to FN in RNase L knockdown cells contributes to increased Rac1 activity and cell migration. 

### 2.9. RNase L Regulates MMP-2 and MMP-9 Activities

**Figure 14:**
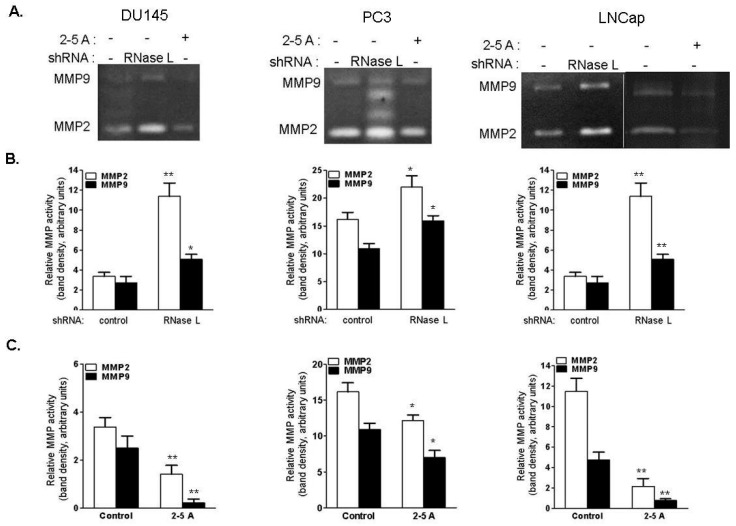
RNase L regulates matrix metalloproteinases (MMP) -2 and MMP-9 activities. (**A**) Gelatin zymography analysis of MMP-2 and MMP-9 activities in conditioned media harvested from DU145, PC3 or LNCaP cells expressing control or RNase L shRNA or transfected with 2–5A complexed with lipofectamine 2000 and added to cells to activate RNase L. Data shown are representative of three independent experiments. Quantitative analysis of MMP-2 and MMP-9 activities in (**B**) Cells expressing RNase L shRNA compared to control shRNA, and (**C**) Cells transfected with 2–5A to activate RNase L compared to control cells. Data shown are mean values ± SEM from three independent experiments. Student’s *t*-test was used to determine *p*-values. * *p* < 0.01, ** *p* < 0.001, and compared to control shRNA expressing cells (**B**); and control cells (**C**).

Matrix metalloproteinases (MMPs) remodel the ECM and play a critical role in cell migration, invasion, tissue metastasis and impact tumor progression [51]. Elevated levels of MMP-2 and MMP-9 are observed in prostate cancer and correlate with increased metastasis [52,53]. To determine if activity of MMP-2 and -9 are regulated by RNase L, culture supernatants of control and RNase L knockdown DU145, PC3 and LNCaP cells were analyzed for activity of secreted MMP-2 and -9 by gelatin zymography. MMP-2 and -9 gelatinase activity, observed by cleared areas on gels, was higher in RNase L-depleted cells than in control cells (1.5–5-fold increase in MMP-2 activity compared to control cells and 1.5–2-fold increase in MMP-9 activity compared to control cells) (Figure 14A,B). Similar increase in MMP-2 and -9 activities was also observed in RNase L KO MEFs compared to WT MEFs (Figure S2). Activation of RNase L by transfecting 2–5A inhibited cell migration (Figure 6E,F) and we observed a decrease in MMP-2 and -9 activity in culture supernatants following 2–5A transfection compared to control cells (Figure 14A,C). Taken together, these results demonstrate that depleting RNase L leads to increased activity of MMP-2 and -9 in prostate cancer cells which correlates with increased cell migration. Silencing of RNase L or activation of RNase L did not affect expression of *MMP-9* mRNA (Figure S3).

## 3. Discussion

In the present study, we demonstrate the role of RNase L as a suppressor of AR signaling, cell migration and activity of matrix metalloproteinases identifying an unrecognized role of RNase L in prostate cancer. Importantly, the nuclease function of RNase L was not required for suppression. The relevance of the HPC1 (*RNASEL*) mutations in prostate cancer is poorly understood. We provide evidence that the most prevalent HPC1-associated mutations in RNase L, R462Q substitution and E265X truncation, enhance AR nuclear translocation, transcriptional activity and cell migration. This study not only identifies Androgen Receptor as a target of RNase L regulation, but also demonstrates that the effect of HPC1-associated mutations on AR signaling and cell migration is amenable to regulation by physiological conditions that exist in prostate cancer cells.

In androgen-treated cells, the interaction between RNase L, Filamin A and AR is disrupted and AR translocates to the nucleus to induce AR-responsive gene expression. In LNCaP cells lacking RNase L significantly higher nuclear AR was observed, similar to FLNA-depleted cells, and cells lacking both proteins showed further increase in nuclear AR which was reflected by increase in AR transcriptional activity, and expression of AR target genes (Figure 1). Increased nuclear AR was only observed in cells treated with R1881 indicating that RNase L effect on AR is ligand-dependent. As would be expected for a dynamic interaction, over-expression of full-length RNase L suppressed AR nuclear localization and AR nuclear activities. In other studies, over-expression of Filamin A inhibited AR transcriptional activity on androgen treatment indicating common underlying mechanisms [27]. Interestingly, the N-terminal fragment of RNase L which interacted with FLNA inhibited AR signaling similar to the full-length protein, while the C-terminal nuclease domain that fails to interact with FLNA, enhanced AR signaling (Figure 2) [3]. Analysis of subcellular AR distribution following R1881 treatment demonstrated increase in nuclear/cytoplasmic ratio in cells lacking RNase L or FLNA which was exacerbated when both proteins were lacking (Figure 3B). Taken together, our data show that RNase L, along with FLNA, may sequester AR in the cytoplasm and in response to R1881 the dissociation of the complex facilitates nuclear localization of AR. In the absence of the ligand, AR remains in the cytoplasm as a part of a multiprotein complex that includes HSP70 and HSP90 [54,55,56]. We have not ruled out the possibility that RNase L may alter AR interaction with HSP proteins. However, given that RNase L has been shown to regulate actin dynamics [3], interact with ECM and cytoskeletal proteins [24,26] and, importantly, inhibition of microtubule and cytoskeletal dynamics inhibits androgen-dependent AR nuclear translocation and AR transcriptional activity [30,31,57,58]; together these data suggest significant involvement of RNase L-regulated actin dynamics in AR translocation.

Sustained signaling through AR is a hallmark of castration-resistant prostate cancer (CRPC) and alternative splicing variants of AR (AR-Vs) that lack ligand-binding domain and constitutively active are reported to be upregulated [59,60,61]. It is established that AR-Vs upregulate transcription of canonical AR-responsive genes and other unique set of target genes [60,62]. Unlike full-length AR, transcriptional activities of the constitutively active AR-Vs are refractory to treatment with taxanes which do not block nuclear translocation of AR-V [63]. Future studies will address if RNase L impacts AR-V-mediated signaling activity.

In addition to transcriptional regulation, AR activity and abundance is regulated at the level of protein degradation and stability [64]. Inhibition of ubiquitin-proteasome degradation pathway has been reported to increase AR levels and several ubiquitin ligases bind to AR and regulate AR functions [65,66,67]. AR levels decreased to similar levels in WT and RNase L knockdown LNCaP cells (Figure 3) in the absence of ligand, R1881, when *de novo* protein translation is inhibited by cycloheximide treatment. Furthermore, inhibiting proteasome-mediated AR degradation by treating cells with MG132 did not result in altered stability of AR protein when RNase L was depleted. Thus, the inhibitory effect of RNase L on AR signaling does not appear to be due to the effect on AR degradation or stability. These observations support the notion that the final activity of AR in any given cell may eventually reflect balance and coordination of several regulators, including RNase L.

HPC1-associated mutations in RNase L have been studied in the context of RNase L enzyme activity and inducing apoptosis [12] as possible explanation for HPC, but do not address how the mutations contribute specifically to prostate cancer. RNase L R462Q mutant had reduced ability to dimerize into an active enzyme and had three-fold reduced activity and E265X produced a truncated protein which lacked the nuclease domain [12]. Since both mutants compromised RNase L enzyme activity, we raised the possibility that nuclease activity may be important. Recent description of RNase L structure allowed identification of residues critical for recognition of 2–5A, dimerization and nuclease function and we designed mutants based on the structural predictions and tested the ribonuclease activity (Figure 4E,F) [37,68]. In addition to the HPC1-associated mutants, we analyzed the RNase L mutants we generated that either lacked enzyme activity due to mutations in nuclease domain (R667A), or have reduced enzyme activity (K166E) or lack activity due to defects in binding activator, 2–5A (Y312A, K240/274N) for effects on AR signaling. The Y312A mutant lacks enzyme activity like the K240/274N and R667A mutations; however it can suppress AR signaling like WT RNase L indicating that enzyme activity may be dispensable (Figure 4). We conclude that in normal prostate cells RNase L is a negative regulator of AR signaling and loss of RNase L function in HPC can enhance AR signaling which is a hallmark of most prostate cancers.

RNase L regulates actin dynamics suggesting a possible role in cell migration [3]. Our results, consistent with other published data [21,22], shows that prostate cancer cells depleted of RNase L show greater migration in wound healing and transwell migration assays in response to fibronectin and serum (Figure 5). In reconstitution experiments, over-expression of RNase L suppressed cell migration compared to both endogenous levels and knockdown cells while activation of RNase L, which requires RNase L dimerization, inhibited cell migration (Figure 6). Unlike WT RNase L, HPC1-associated mutants, R462Q and E265X supported enhanced cell migration. Other RNase L mutants which have reduced activity (K166E) or lack activity (R667A and K240/274N) show enhanced cell migration. RNase L Y312A is an exception in that it lacks enzyme activity but suppresses cell migration like WT RNase L. The difference in the effect of K240/274N and Y312A may reflect the roles each of the residues play in contacting 2–5A versus disrupting domain interactions. K240 and K274 are critical residues in the P-loop motifs in the ankyrin repeats and may disrupt ankyrin repeat-protein kinase domain interaction and prevent 2–5A binding thereby affecting RNase activity [69]. It is possible that K240/274N mutant alters binding of proteins that contact RNase L through ankyrin repeats and the pseudokinase domains. Y312 is one of the residues that have been shown to provide direct contact with 2–5A and Y312A substitution may therefore lack activity while possibly retaining folding [37]. The precise mechanism by which these mutants affect interactions with other proteins will be addressed in future studies.

The mutations we have tested for AR signaling and cell migration span all the functional domains of RNase L. Mutations in the N-terminal ankyrin repeat domain, which serves as protein interaction domain, have variable effects on nuclear AR and cell migration and mutation in the C-terminal catalytic domain fails to suppress like WT. Biochemical studies showed that RNase L associated with the cytoskeleton assumes an inactive conformation and exists as a monomer while retaining the ability to bind with interacting proteins [24,70]. 2–5A binds to ankyrin repeat 2 and 4 and the pseudokinase domain facilitating dimerization and enzymatic activation. Activation of RNase L may cause dramatic conformational change and dimerization that induces its release from interacting proteins. Based on our results, we propose that the effect of RNase L on AR signaling and cell migration is mediated, in part, by protein-protein interactions and does not require enzymatic activity. Several proteins like Filamin A, LNX and or other cytoskeletal proteins may contribute to both these effects by interacting with RNase L [3,24,26]. Additional studies are required to investigate if the effect of the RNase L mutants is mediated by altered interaction with any of these interacting proteins. RNase L also inhibits cell attachment to ECM substrates and cell spreading on fibronectin. An earlier report showed that activity of RNase L downregulates transcripts involved in cell adhesion both transcriptionally and post-transcriptionally [22]. In contrast, we observed the suppressive effect of RNase L on cell migration in cells expressing endogenous RNase L in the absence of activation or with mutants that lacked enzyme activity.

We explored the underlying mechanisms for increased cell migration in RNase L-depleted cells by analyzing activation of integrin β1 which is primarily involved in adhesion to fibronectin. Increased cell surface expression of integrin β1 in RNase L-depleted cells corresponded with increase in cell migration and decrease correlated with activation of RNase L and inhibition of cell migration (Figure 9 and Figure 10). Further, in androgen responsive LNCap cells, treatment with R1881 resulted in increase in cell surface expression of integrin β1 and RNase L-depleted cells treated with R1881 showed a corresponding increase in integrin β1 expression on cell surface (Figure S4). Activated integrins recruit and induce phosphorylation of FAK and Src which initiate signaling events to activate Rho GTPases to control cell shape and motility [41,42,47]. Accordingly, increase in integrin-stimulated FAK and Src phosphorylation was observed in RNase L-depleted cells and RNase L KO MEFs in response to fibronectin. Inhibiting Src activity with PP2 inhibitor in RNase L-depleted cells decreased cell migration compared to mock-treated cells demonstrating the involvement of the pathway for cell migration. Activity of Rho GTPases is required for cell migration and our testing of small Rho GTPases revealed that expression of dominant-negative form of Rac1 (T17N) significantly inhibited migration of RNase L-depleted cells, whereas dominant-negative Cdc42 (T17N) or RhoA (T19N) did not. Rac1 activity is regulated by Src in many cell types and elevated expression of Rac1 is observed in prostate cancer cells and tumors [71,72]. Our results show higher activity of Rac1 in response to fibronectin in RNase L-depleted cells compared to control cells although total levels of Rac1 protein were very similar. This is the first evidence of regulation of Rac1 activity by RNase L. Interestingly, the two proteins that interact with RNase L, namely, Filamin A and IQGAP1 are reported to regulate Rac1 activity [73]. Based on these observations and published data, RNase L appears to regulate cytoskeletal events, including AR signaling and cell migration, by virtue of its association and interaction with cytoskeletal and motor assembly proteins [26]. Apart from effects on cell migration, RNase L regulates activity of MMP-2 and MMP-9. Knockdown of RNase L increases gelatinase activity of both MMP-2 and -9 (Figure 14) which can enhance ECM remodeling and invasive potential of cells. Consistent with our observations, activation of RNase L inhibited integrin activation, cell migration as well as activity of MMP-2 and -9. MMP activities can be regulated at the level of transcription and post-transcriptionally by activators and inhibitors [74]. Levels of *MMP-9* mRNA did not change in knockdown cells and while not explored here, RNase L may affect MMP activity by regulating natural MMP inhibitors, tissue inhibitors of metalloproteinases (TIMPs).

Collectively, the results presented here identify RNase L as a suppressor of AR signaling, cell migration and MMP activity. Using RNase L mutants we show that nucleolytic activity is dispensable for both AR signaling and migration. HPC1-associated mutations in RNase L enhance AR signaling and cell migration and our study has identified a novel role of RNase L as a prostate cancer susceptibility gene. It is likely that RNase L mutations identified as risk factors in other types of cancers may affect cell migration and contribute to tumor development extending the antitumor role of RNase L beyond prostate cancer.

## 4. Materials and Methods 

### 4.1. Chemicals, Reagents and Antibodies

Chemicals, unless indicated otherwise, were from Sigma Aldrich (St. Louis, MO, USA). Synthetic androgen R1881 was from Sigma Aldrich, extracellular matrices fibronectin, vitronectin, laminin, collagen I, collagen IV and Src inhibitor PP2 were from Millipore (Billerica, MA, USA). Cycloheximide (Sigma-Aldrich) and MG132 (EMD-Millipore, Billerica, MA, USA) were used at indicated concentrations. Antibodies to AR (N-20), Integrin β1 (M-106) and Filamin A were from Santa Cruz Biotechnology (Santa Cruz, CA, USA), Rac-1 (clone 102) was from BD Biosciences (San Jose, CA, USA). Total FAK, phospho-FAK (Y397), total Src, phospho-Src (Y416) were from Cell Signaling, Inc. (Danvers, MA, USA). RNase L monoclonal antibody was kindly provided by Robert Silverman (Cleveland Clinic). Antibodies to β-actin, monoclonal and polyclonal antibodies to Flag tag, Flag-M2 agarose beads were from Sigma Aldrich. Anti-mouse IgG and anti-rabbit IgG HRP linked secondary antibodies were from Cell Signaling, Inc. (Danvers, MA, USA) and ECL reagents were from GE Healthcare (Piscataway, NJ, USA) and Boston Bioproducts (Ashland, MA, USA). Alexa 488-labeled Phalloidin, and Alexa fluor 647 donkey anti-rabbit IgG were from Life Technologies, Carlsbad, CA, USA. 

### 4.2. Cell Culture and Transfections

DU145, PC3 cells, WT and Rnasel^−/−^ MEFs ((RNase L KO) primary and transformed with SV40 large T antigen, kindly provided by R.H. Silverman, Cleveland Clinic) and LNCaP cells (ATCC, Manassas, VA, USA) were grown in Roswell Park Memorial Institute (RPMI) 1640 supplemented with 100 μg/mL penicillin/streptomycin, 2 mM l-glutamine and 10% heat-inactivated fetal bovine serum (Sigma-Aldrich, St. Louis, MO, USA). For experiments involving androgen treatment, cells were transferred to phenol red-free medium supplemented with 2% charcoal-stripped serum (Hyclone, Logan, UT, USA) at least 24 h prior to addition of the synthetic androgen R1881 (1 nM) or ethanol vehicle (0.01%). Cells were maintained in 95% air, 5% CO_2_ at 37 °C. RNase l-silencing (targeting the 3′-UTR), Filamin-A silencing, and non-silencing shRNAs were generated as suggested by the manufacturer using a GIPZ-lentiviral shRNA system and knock down cells or controls were selected with 1 μg/mL of puromycin as described previously [3] (Open Biosystems, Thermo Scientific, PA, USA). In some experiments, RNase L was knocked down using shRNA plasmid as described previously [16]. Knock-down of endogenous proteins was determined by western blotting. Transfection of 2–5A (10 μM) was performed using lipofectamine 2000 (Invitrogen, Thermo Fisher Scientific, Waltham, MA, USA) according to the manufacturer’s protocol as described previously [75]. Briefly, cells were plated 1 day before transfection, so that the cells are 80%–90% confluent at the time of transfection. 2–5A was diluted into serum-free media and then mixed with lipofectamine 2000 reagent for 15 min before being added to cells in growth media. Preparation of 2–5A using ATP and recombinant 2–5A synthetase (a generous gift from Rune Hartmann, University of Aarhus, Aarhus, Denmark) has been described previously [75]. PolyI:C (2 μg/mL) was transfected into cells using Polyjet reagent (SignaGen Laboratories, Gaithersburg, MD, USA). Activity of RNase L in intact cells was determined in HeLa cells reconstituted with Flag-RNase L mutant constructs as described previously [75]. In experiments involving inhibitors, cells were preincubated with inhibitor for 1 h prior to treatment and then replaced with growth medium.

### 4.3. Plasmids

Plasmids Flag-RNase L, Flag-RNase L R667A, Flag-RNase L (1–335, ΔC), Flag-RNase L (386–741, ΔN) (kindly provided by Robert Silverman, Cleveland Clinic) were transfected using lipofectamine 2000 as per manufacturer’s instructions. The Flag-RNase L mutants were constructed by site-directed mutagenesis using the primers listed in Table 1 and QuikChange Lightning Multi Site-Directed Mutagenesis Kit (Agilent Technologies, Santa Clara, CA, USA). The constructs were sequenced to confirm the mutations and expression confirmed by immunoblot analysis. The dominant negative Myc-tagged RhoA (T19N), Rac1 (T17N) and Cdc42 (T17N) eukaryotic expression constructs were described previously [50].

### 4.4. Co-Immunoprecipitation and Immunoblotting

LNCaP cells expressing Flag-RNase L plasmid were either not treated, treated with R1881 (1 nM) for 1 h or 24 h and harvested. Cells were washed with ice cold PBS and lysed in buffer containing 0.5% NP-40, 90 mM KCl, 5 mM magnesium acetate, 20 mM Tris, pH 7.5, 5 mM β mercaptoethanol, 0.1 M phenylmethylsulfonyl fluoride (PMSF), 0.2 mM sodium orthovanadate, 50 mM NaF, 10 mM glycerophosphate, protease inhibitor (Roche Diagnostics, Indianapolis, IN, USA) on ice for 20 min. The lysates were clarified by centrifugation at 10,000× *g* (at 4 °C for 20 min). Clarified cell lysates were precleared and mixed with control IgG or FlagM2-agarose beads and rotated end-to-end 1 h or overnight at 4 °C. The beads were collected and washed five times in lysis buffer. The immunoprecipitated proteins were dissociated by boiling in Laemmli sample buffer, separated on 10% SDS-polyacrylamide gels, transferred to nitrocellulose membrane (Biorad, Hercules, CA, USA) and subjected to immunoblotting. Membranes were probed with different primary antibodies according to the manufacturer’s protocols. Membranes were washed with Tris Buffered Saline (TBS) with 1% Tween 20 and incubated with goat anti-mouse or goat anti-rabbit antibody tagged with horseradish peroxidase (Cell Signaling, Danvers, MA, USA) for 1 h. Proteins in the blots were detected by enhanced chemiluminesence (GE Healthcare). Cell extracts from LNCaP cells treated with cycloheximide (50 μg/mL) alone or combined with MG132 (20 μM) or R1881 (1 nM) were prepared in 2% sodium dodecyl sulphate (SDS) and subjected to immunoblotting. PC3 (control and RNase L knockdown) cells or WT and RNase LKO MEFs were serum starved for 16 h and plated on fibronectin coated dishes for 1 h. FAK and Src phosphorylation was detected by immunoblotting using anti-phospho FAK (Y397) or anti-phospho Src (Y416) antibodies (Cell Signaling, Danvers, MA, USA).

### 4.5. Immunofluorescence Assays

LNCaP cells (WT, RNase L knockdown, Filamin A knockdown or RNase L and Filamin A knockdown) on glass coverslips were treated with phenol red-free RPMI medium supplemented with 2% charcoal-stripped serum (Hyclone, Logan, UT, USA) at least 24 h prior to addition of the synthetic androgen R1881 (1 nM) or ethanol vehicle (0.01%) for 24 h. In some experiments, LNCaP RNase L-knockdown cells were reconstituted with Flag-RNase L (WT) or various Flag-RNase L mutants and plated on coverslips and treated as described above. Cells were fixed in 4% paraformaldehyde (Boston Bioproducts, MA, USA) for 15 min and permeabilized with 5% goat serum, 0.3% Triton-X-100 in PBS for 1 h. After washing and blocking in 1% Bovine Serum Albumin (BSA) in PBS, the cells were reacted with anti-AR antibody (N-20, Santa Cruz Biotechnology, CA, USA, 1:200 in 1% BSA, 0.3% Triton-X-100 in PBS) at 4 °C for 16 h followed by washing and incubation with fluorescent dye-conjugated secondary antibodies, Alexa-647 Goat anti-rabbit IgG (1:200, 1 h at 4 °C, Molecular probes, CA, USA). Cells were mounted in Vectashield with DAPI to stain the nucleus (Vector Labs, Burlingame, CA, USA). Fluorescence and confocal microscopy assessments were performed with Leica CS SP5 multi-photon laser scanning confocal microscope (Leica Microsystems, Weitzler, Germany) and quantitated using Image J software (National Institutes of Health). Images were processed using Adobe Photoshop CS4 (Adobe, San Jose, CA, USA). More than 10 cells (from at least three fields) were analyzed for each condition from three independent experiments.

### 4.6. Luciferase Reporter Gene Assays

LNCaP cells (WT, RNase L knockdown, Filamin A knockdown or RNase L and Filamin A knockdown) were transfected with AR-responsive PSA-luciferase [76] (1.0 μg), and plasmid pCH110 expressing β-galactosidase (0.1 μg) to normalize transfection efficiency in phenol red-free RPMI medium supplemented with 2% charcoal-stripped serum (Hyclone, Logan, UT, USA) at least 24 h prior to addition of the synthetic androgen R1881 (1 nM) or ethanol vehicle (0.01%). Cells were harvested in luciferase lysis buffer 24 h after treatment, and luciferase activity was determined using luciferase assay kit (Promega, Madison, WI, USA) and normalized to β-galactosidase levels. Experiments were performed in triplicate, and the results are representative of three independent experiments and shown as ± SD.

### 4.7. RNA Isolation and Quantitative Real Time Polymerase Chain Reaction

RNA was isolated using Trizol reagent (Invitrogen, Thermo Fisher Scientific,) as per the manufacturer’s instructions and used for cDNA synthesis using random decamers and a RETROscript cDNA synthesis kit (Life Technologies; Thermo Fisher Scientific). Expression of androgen-responsive genes was determined by quantitative reverse transcription polymerase chain reaction (qRT-PCR) using SYBR Green PCR Master Mix (Bio-Rad Laboratories Inc., Hercules, CA, USA) using the gene-specific primers and normalized to *GAPDH* expression. Primer sequences used are listed in Table 2 below. Experiments were performed in triplicate, and the results are representative of three independent experiments and shown as ± SD.

### 4.8. Cell Fractionation

LNCaP cells (WT, RNase L knockdown, Filamin A knockdown or RNase L and Filamin A knockdown) were treated with R1881 or vehicle for 24 h and harvested. Twenty-five percent of the cells were saved as input, and the remaining portion was fractionated into nuclear and cytosolic fractions using Nuclear/Cytosol Fractionation Kit (MBL International Corp., Woburn, MA, USA). The fractions were then subjected to immunoblotting as described above to measure AR levels and quantitated using NIH Image J software. 

### 4.9. Wound Healing Assay

Confluent monolayers of cells in 6-well plates were incubated in serum-free medium for 24 h. Monolayers were scratched with a micropipette tip and washed in phosphate buffer saline (PBS), and replaced with complete growth medium. Migration of cells to close the wound was monitored at indicated times and phase-contrast images were acquired on an Olympus IX81 inverted microscope using a 10× objective lens and a XM10 camera (Olympus, Tokyo, Japan). Wound closure was calculated from at least three independent experiments using NIH Image J software. Data shown are representative of three independent experiments and quantitation is shown as ± SD.

### 4.10. Cell Migration Assay

Transwell cell migration assays were performed using a modified Boyden chamber (Corning Inc., Corning, NY, USA) containing a fibronectin-coated polycarbonate membrane filter (6.5 mm diameter, 8 µm pore size) in growth medium. DU145, PC3 or LNCaP cells (2 × 10^5^) cells (control or RNase L knockdown) and WT or RNase L KO MEFs were incubated in serum-free medium for 24 h and plated in the upper chamber and allowed to migrate for indicated times to lower chamber that contained growth medium with 10% fetal bovine serum (FBS). Non-migrated cells on the upper chamber were scraped with a cotton swab, and migrated cells on the bottom surface were trypsinized and counted with a hemocytometer. Experiments were performed in triplicate, and the results are representative of three independent experiments and shown as mean ± SEM.

### 4.11. Cell Spreading Assay

DU145 or PC3 (control or RNase L-knockdown) were plated on fibronectin-coated coverslips for indicated times up to 100 min. Attached and spread cells were fixed with 3.7% paraformaldehyde in PBS (Boston Bioproducts, Ashland, MA, USA) for 10 min and permeabilized with 0.1% Triton-X-100 for 5 min. F-actin was labeled with Alexa 488-labeled phalloidin (Life Technologies, CA, USA) and mounted in Vectashield with DAPI (4′,6-diamidino-2-phenylindole, Vector Laboratories, Burlingame, CA, USA). Cells were imaged by the use of a Leica TCS SP5 multiphoton laser scanning confocal microscope (Leica Microsystems, Weitzler, Germany), and cell area was quantitated using NIH Image J software. Experiments were repeated in triplicate with at least 30 measurements per time point for each experiment. Data are shown as mean area at indicated time points ± SEM. 

### 4.12. Cell Attachment Assay

To quantitate cell attachment to different extracellular matrix substrates, 96-well plates were coated with 10 μg/mL each of fibronectin, vitronectin, laminin, collagen I or collagen IV (EMD Millipore, Billerica, MA, USA). Prior to use, the wells were treated with 1% BSA in 1× PBS (pH 7.4). Cells (2 × 10^5^) in single cell suspension in serum-free medium were added per well in triplicate and incubated at 37 °C for 1 h. Wells were washed three times with PBS, fixed in 95% ethanol and stained with 0.1% crystal violet for 30 min at room temperature. The wells were washed extensively to remove excess stain. Cells were lysed in 0.2% Triton-X-100 and absorbance measured at 570 nm. Percent cell attachment was determined from three independent experiments performed in triplicate and shown as mean ± SEM.

### 4.13. Flow Cytometry and Analysis

DU145, PC3 or LNCaP cells (control or RNase L-knockdown) were grown in 100 mm dishes to 80%–90% confluence and then plated on dishes coated with 10 μg/mL fibronectin for 2 h. Cells were harvested and resuspended in ice-cold HEPES-buffer (20 mM HEPES, 125 mM NaCl, 45 mM glucose, 5 mM KCl, 0.1% albumin, pH 7.4) and incubated with anti-integrin β1 antibodies (Santa Cruz Biotechnologies, Santa Cruz, CA, USA) for 1 h. Isotype-specific antibodies were used as controls. Cells were washed three times in HEPES-buffer and incubated with Alexa-fluor-488 labeled secondary antibodies. Flow cytometry was performed using a FACSCalibur System (BectonDickinson, Heidelberg, Germany) equipped with Cell Quest software (Becton-Dickinson, San Jose, CA, USA). In some experiments, cells were transfected with 2–5A (10 μM) for 4 h and subject to flow cytometry analysis.

### 4.14. Rac Activity Assays 

DU145 or PC3 cells (control and RNase L knockdown) were plated on dishes coated with 10 μg/mL fibronectin for 30 min or 1 h. Assays for GTP-bound Rac1 were performed as described [77]. Cells were lysed and precipitated using GST-PBD (PAK-binding domain) beads for pulling down active Rac1. Bead bound proteins (for active Rac1) and cell lysates (total Rac1) were resolved on a 13% SDS-PAGE gel and transferred onto nitrocellulose membranes and probed with primary antibodies against Rac1. The levels of active Rac1 were calculated by comparing the intensities of the active Rac1 bands with those of the total Rac1 bands in each lane using Image J software (National Institutes of Health). Data are expressed as fold increase in Rac1 activity over control and are representative of three independent experiments. 

### 4.15. Gelatin Zymography

Gelatin zymography was performed under non-reducing conditions on 8% polyacrylamide gels copolymerized with 0.1% gelatin (Sigma-Aldrich, St. Louis, MO, USA). Activity of MMP-9 and -2 was determined in culture supernatants of control and RNase L knockdown cells (2 × 10^5^ cells) treated or not with 2–5A (10 μM) as described previously [78]. Clear bands representing MMP-9 and -2 activities were imaged and quantitated using Image J software (National Institute of Health). Data shown are representative of three independent experiments and quantitation is shown mean as ± SD.

### 4.16. Statistical Analysis 

All values are presented as mean ± SEM from at least three independent experiments or are representative of three independent experiments performed in triplicate and shown as mean ± SD. Student’s *t*-tests were used for determining statistical significance between groups. *p-*values are shown for all experiments and *p* < 0.05 was considered significant.

## 5. Conclusions

In this study, we demonstrate a role of RNase L as a suppressor of Androgen Receptor (AR) signaling, cell migration and matrix metalloproteinase activity. Using RNase L mutants, we show that its nucleolytic activity is dispensable for both AR signaling and migration. The most prevalent HPC1-associated mutations in RNase L, R462Q and E265X, enhanced AR signaling and cell migration and our studies identify a novel role of RNase L as a prostate cancer susceptibility gene.

## Figures and Tables

**Table 1:** List of primers used for mutagenesis.

Primer Name	Sequence of Primer
K274N R	5′-CAAGCAGCAGTGCTGT*A*TTGCCATCACTGTCTGTG-3′
K274N F	5′-CACAGACAGTGATGGCAA*T*ACAGCACTGCTGCTTG-3′
K240N R	5′-GGATCAGGGGAGT*A*TTCCCTCTTTCTCCCC-3′
K240N F	5′-GGGGAGAAAGAGGGAA*T*ACTCCCCTGATCC-3′
R667A R	5′-CAATGTGTTCTCCCAAATTC*GC*GATGAACTTTAGCAGATCAC-3′
R667A F	5′-GTGATCTGCTAAAGTTCATC*GC*GAATTTGGGAGAACACATTG-3′
R462Q R	5′-TAAATATAGATGACAGGACATTT*T*GGGCAAATTCATCTTCCTCATTT-3′
R462Q F	5′-AAATGAGGAAGATGAATTTGCCC*A*AAATGTCCTGTCATCTATATTTA-3′
E265X R	5′-CTGTGTCATTAATCT*A*TATGTGCTCTTGCTCCAGAAGC-3′
E265X F	5′-GCTTCTGGAGCAAGAGCACATA*T*AGATTAATGACACAG-3′
K166E F	5′-AGAGCGGCTGAGG*G*A*G*GGAGGGGCCACAG-3′
K166E R	5′-CTGTGGCCCCTCC*C*T*C*CCTCAGCCGCTCT-3′
Y312A R	5′-CAAGGGAATGGTCA*GC*ATTCCGCCTCGCTGTCATAACAAGAT-3′
Y312A F	5′-ATCTTGTTATGACAGCGAGGCGGAAT*GC*TGACCATTCCCTTG-3′

**Table 2:** List of primers for quantitative real-time polymerase chain reaction.

Primer Name	Sequence of Primer
PSA F	5′-GCAGCATTGAACCAGAGGAG-3′
PSA R	5′-CCCATGACGTGATACCCTGA-3′
sGCα1 F	5′-CTGCCTCATTTGCTTCATCA-3′
sGCα1 R	5′-TTGCCATGCTGAGCTGTTTA-3′
ETV1 F	5′-CACTGGGTCGTGGTACTCCT-3′
ETV1 R	5′-TACCCCATGGACCACAGATT-3′
MMP9 F	5′-GCCATTCACGTCGTCCTTAT-3′
MMP9 R	5′-TTGACAGCGACAAGAAGTGG-3′

## Supplementary Materials

Supplementary materials can be found at www.mdpi.com/1422-0067/18/3/529/s1.
