# Supplementary material for: RNase L Suppresses Androgen Receptor Signaling, Cell Migration and Matrix Metalloproteinase Activity in Prostate Cancer Cells
Source: Int J Mol Sci. 2017 Mar 1;18(3):529. doi: 10.3390/ijms18030529 (PMC5372545; doi:10.3390/ijms18030529)
Supplement: Supplementary file 1 [file ijms-18-00529-s001.pdf]

Supplementary Materials: RNase L Suppresses Androgen Receptor Signaling, Cell Migration and Matrix Metalloproteinase Activity in Prostate Cancer Cells

Shubham Dayal, Jun Zhou, Praveen Manivannan, Mohammad Adnan Siddiqui, Omaima Farid Ahmad, Matthew Clark, Sahezeel Awadia, Rafael Garcia-Mata, Lirim Shemshedini and Krishnamurthy Malathi

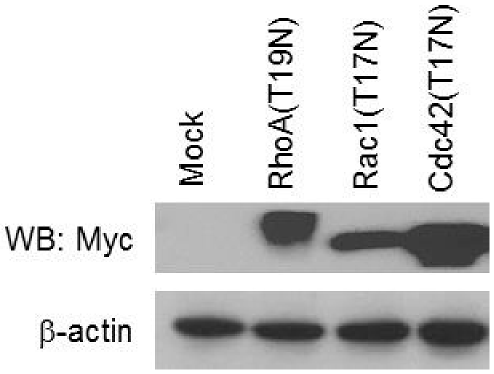

Figure S1. Expression of RhoA (T19N), Ras-related C3 botulinum toxin substrate 1-guanosine—Rac1 (T17N) and cell division control protein 42 homolog—Cdc42 (T17N) in PC3 cells compared to cells receiving vector alone (mock). Cell lysates were analyzed by immunoblot analysis using anti-Myc antibody and normalized to β-actin levels.

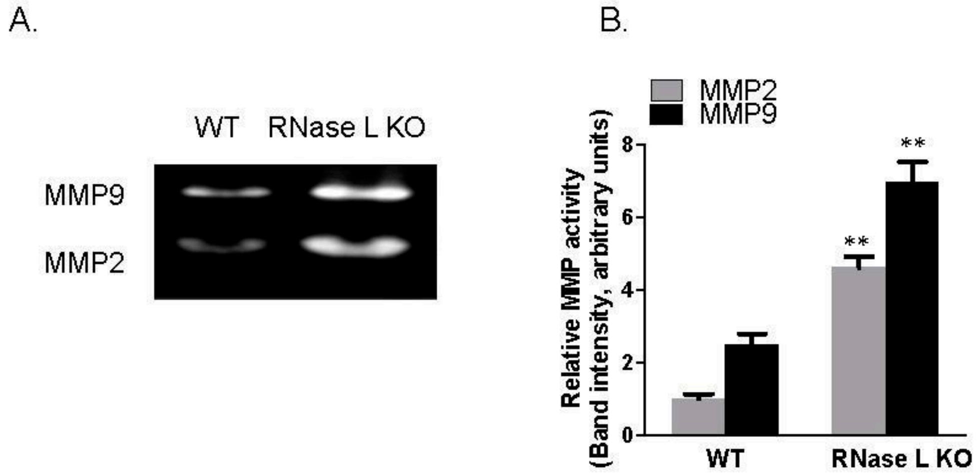

Figure S2. Increased matrix metalloproteinase (MMP)-2 and MMP-9 activities in RNase L KO mouse embryonic fibroblasts (MEFs). (**A**) Gelatin zymography analysis of MMP-2 and MMP-9 activities in conditioned media harvested from Wild type (WT) and RNase L KO MEFs. Data shown is representative of three independent experiments. (**B**) Quantitative analysis of MMP-2 and MMP-9 activities in WT and RNase L KO MEFs. Data shown are mean values ± standard error of mean (SEM) from three independent experiments. Student's *t*-test was used to determine *p*-values. * $p < 0.01$, ** $p < 0.001$, and compared to control WT MEFs.

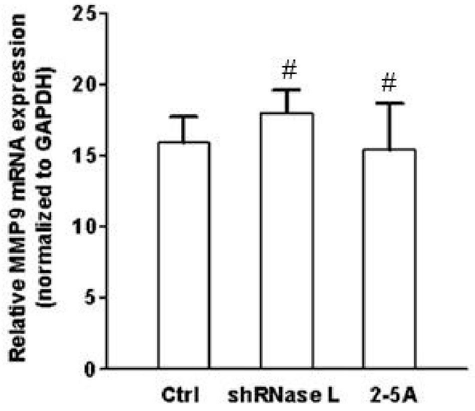

Figure S3. Effect of RNase L on *MMP-9* mRNA levels. Reverse transcriptase-polymerase chain reaction (RT-PCR) analysis of messenger RNA (mRNA) levels in DU145 cells expressing control or RNase L short hairpin RNA (shRNA) or transfected with 2–5A complexed with lipofectamine 2000 and added to cells to activate RNase L normalized to glyceraldehyde-3-phosphate dehydrogenase (*GAPDH)* mRNA levels. Student's *t* test was used to determine *p* values. # not significant, and compared to control cells.

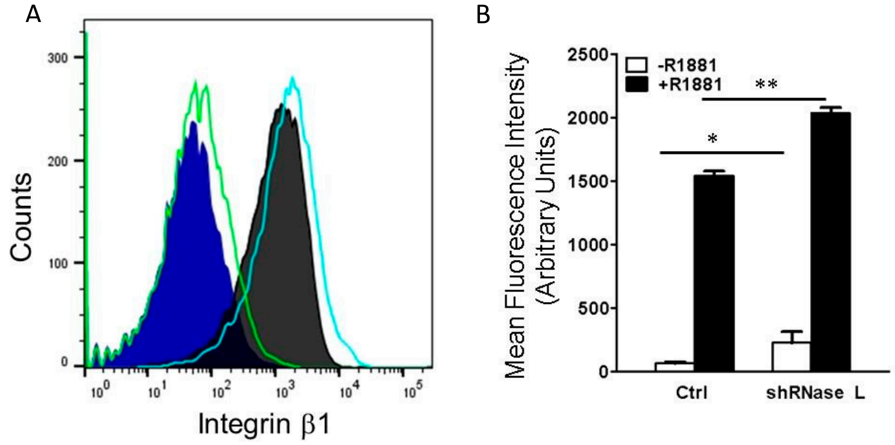

Figure S4. Effect of androgens on cell surface expression of integrin β1. LNCaP cells expressing control shRNA or RNase L shRNA were treated with R1881 (1 nM) and analyzed by flow cytometry for surface staining with antibodies against integrin β1 and Alexa-488 conjugated secondary antibodies. (**A**) Representative histograms, and (**B**) Bar graphs for the mean fluorescence intensity of at least three independent experiments for integrin β1 are shown. Student's *t*-test was used to determine *p*-values. * $p < 0.01$, ** $p < 0.001$ and compared to cells expressing control shRNA; Ctrl: control.
